# Ethyl pyruvate improves white matter remodeling in rats after traumatic brain injury

**DOI:** 10.1111/cns.13534

**Published:** 2020-12-27

**Authors:** Leilei Mao, Limin Sun, Jingyi Sun, Baoliang Sun, Yanqin Gao, Hong Shi

**Affiliations:** ^1^ Department of Neurology Second Affiliated Hospital Key Laboratory of Cerebral Microcirculation at the University of Shandong Shandong First Medical University & Shandong Academy of Medical Sciences Taian China; ^2^ State Key Laboratory of Medical Neurobiology MOE Frontiers Center for Brain Science and Institute of Brain Sciences Fudan University Shanghai China; ^3^ Department of Anesthesiology of Shanghai Pulmonary Hospital Tongji University Shanghai China

**Keywords:** ethyl pyruvate, microglia, traumatic brain injury, white matter injury

## Abstract

**Background:**

Severe traumatic brain injury (TBI) results in long‐term neurological deficits associated with white matter injury (WMI). Ethyl pyruvate (EP) is a simple derivative of the endogenous energy substrate pyruvate with neuroprotective properties, but its role in recovery from WMI has not been explored.

**Aims:**

This study examines the effect of EP treatment on rats following TBI using behavioral tests and white matter histological analysis up to 28 days post‐injury.

**Materials and Methods:**

Anaesthetised adult rats were subjected to TBI by controlled cortical impact. After surgery, EP or Ringers solution (RS) was administrated intraperitoneally at 15 min after TBI and again at 12, 24, 36, 48, and 60 h after TBI. Sensorimotor deficits were evaluated up to day 21 after TBI by four independent tests. Immunofluorescence and transmission electron microscopy (TEM) were performed to assess white matter injury. Microglia activation and related inflammatory molecules were examined up to day 14 after TBI by immunohistochemistry or real‐time PCR.

**Results:**

Here, we demonstrate that EP improves sensorimotor function following TBI as well as improves white matter outcomes up to 28 d after TBI, as shown by reduced myelin loss. Furthermore, EP administration during the acute phase of TBI recovery shifted microglia polarization toward the anti‐inflammatoryM2 phenotype, modulating the release of inflammatory‐related factors.

**Conclusion:**

EP treatment may protect TBI‐induced WMI via modulating microglia polarization toward M2.

## 1 INTRODUCTION

Traumatic brain injury (TBI) is a major public health concern owing to its high mortality and morbidity. Globally, more than 60 million new cases of TBI occur every year, and around 10 million people die every year after TBI.^1^ Survivors of TBI frequently suffer from long‐term motor and cognitive dysfunction.^2^, ^3^ White matter injury (WMI) is a significant consequence of TBI and is associated with worsened functional outcomes post‐TBI in preclinical models.^4^ Accumulating neuroimaging data has shown that WMI is strongly correlated with worsened neurological outcomes in TBI patients.^5^, ^6^, ^7^ However, no effective therapies currently protect against WMI in TBI patients as of yet.

Although WMI may focus on the loss of integrity to the myelin structure afforded by oligodendrocytes, the injury itself can be amplified by pro‐inflammatory elements in the damaged tissue. The loss of white matter integrity leads to fragmentation of the myelin sheath, leaving myelin debris in the extracellular milieu. In the process of recovery, phagocytosis of myelin fragments by phagocytic cells may be beneficial toward the restoration of myelination and functional recovery. To this end, the shift in polarization of microglia/macrophage from the pro‐inflammatory M1 phenotype to the antiinflammatory M2 phenotype is thought to aid in phagocytosis of toxic myelin fragments and support restoration and recovery from WMI.

Ethyl pyruvate (EP) is a stable lipophilic ester derivative of pyruvate that is reported to be remarkably effective in protecting against traumatic or ischemic cerebral injury.^8^, ^9^, ^10^, ^11^, ^12^ However, few studies have addressed whether protective effect of EP impacts WMI after TBI, and little is known about the underlying mechanisms. Therefore, this study examines the effect of EP treatment on rats following TBI using behavioral tests and white matter histological analysis up to 28 days postinjury. Our results suggest that the effect of EP treatment on white matter remodeling and the modulation of microglia polarization is a potential mechanism underlying EP‐afforded neuroprotection.

## 2 MATERIALS AND METHODS

### 2.1 Experimental design

**FIGURE 1:**
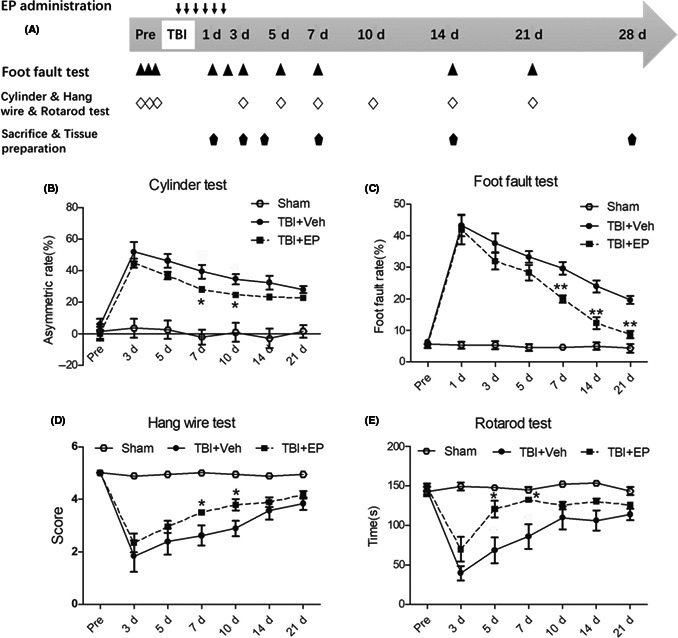
Effects of EP treatment on sensorimotor function following CCI. (A) Flow chart for the experimental design. (B) Asymmetry of forelimb usage for postural weight support was assessed in rats 3–21 days post‐CCI or sham surgery. (C) The number of foot faults expressed as a percentage of total steps was assessed to reflect the fidelity of descending sensorimotor pathways 1–21 days following CCI. (D) Rats were scored on their response to placement on the hang wire test 3–21 days following CCI. (E) Rats were subjected to rotarod testing for balance/proprioception 3–21 days post‐CCI. “Pre” represents presurgery baseline function. Data are mean ± SE, *n* = 10 animals/group. *p < 0.05, **p < 0.01 vs. vehicle‐treated TBI animals

This study was approved by the Institutional Animal Care and Use Committee of the Shandong First Medical University and performed in accordance with the NIH *Guide for the Care and Use of Laboratory Animals*. Adult male Sprague Dawley rats (10–12 weeks old, 280–320 g) were purchased from Pengyue Laboratory Animal Breeding Co., Ltd (Jinan, China) and housed under standard laboratory conditions (temperature 22 ± 1°C; humidity 50 ± 10%), with a 12‐h light‐dark cycle and access to food and water for 1‐2 weeks prior to experimentation. The rats were randomized into three groups after 1 week of acclimation: sham group treated with the vehicle (Ringers solution, RS) (sham), TBI group treated with RS (TBI + Veh), and TBI group treated with EP (TBI + EP). After surgery, EP or RS was administrated intraperitoneally at 15 min after TBI and again at 12, 24, 36, 48, and 60 h after TBI. Behavioral tests were carried out at up to day 21 after TBI. Rats were sacrificed for sample collection, immunofluorescence, or transmission electron microscopy (TEM) from 128 days following the surgery. The flow chart for the experimental design is shown in Figure 1A.

### 2.2 Surgical procedures

The rats were initially anesthetized with 4% isoflurane and then maintained with 1.5%–2.5% isoflurane during surgery. Animals were fixed on a stereotaxic frame with the head positioned in a horizontal plane. All surgical procedures were performed under general anesthesia and have been previously described in detail.^8^ In brief, following a midline incision, a right parietal craniotomy (2.7 mm frontal and 2.7 mm lateral to the bregma, diameter 5.4 mm) was made with a drill. Unilateral controlled cortical impact (CCI) injury was produced using a pneumatically driven cortical impact device (TBI 0310; Precision Systems and Instrumentation, Fairfax Station, VA, USA) with a circular impactor tip (5 mm diameter). A moderate severity of CCI injury was induced, using 4.0 m/second velocity and 3.2 mm depth of tissue compression for 0.5 s. Sham injury rats were anesthetized and underwent similar procedures to control for surgical stress, but sham rats did not receive any impact. Animals were placed to their home cages after the surgery.

### 2.3 EP administration

Ethyl pyruvate (Sigma‐Aldrich, St. Louis, MO, USA) was freshly prepared in 1 mL RS containing Na^+^ (130 mmol/L), K^+^ (4 mmol/L), Ca^2+^ (2.7 mmol/L), and Cl^‐^ (139 mmol/L). Rats were randomly assigned to receive intraperitoneal injections of either 30 mg/kg of EP or equal volume of vehicle (1 mL RS) at 15 min after CCI and again at 12, 24, 36, 48, and 60 h.

### 2.4 Behavioral testing

Neurological functional deficits were assessed with the cylinder, foot fault, rotarod, and hang wire tests by three different researchers who were blinded to the study. The rats were subjected to a variety of somatosensory and motor tests before and after surgery. All behavior tests were repeated 3 days before CCI for training. All outcome assessments were carried out by investigators blinded to treatment assignments.

#### 2.4.1 Hang wire test

The wire‐hanging apparatus included 2 vertical supports (45 cm high) and a thin steel bar (50 cm length; 3 mm diameter) resting on a flat surface. Rats were placed in a hanging position from the steel bar and were observed for 30 s in 3 trials. The behavior of the rats while hanging from the thin bar was recorded and scored according to the following system: 0, fell off; 1, hung onto the bar with both forepaws; 2, hung onto the bar with added attempt to climb onto the bar; 3, hung onto the bar with both forepaws and one or both hind paws; 4, hung onto the bar with all four paws and with tail wrapped around the bar; 5, escaped to either of the side supports.

#### 2.4.2 Rotarod test

The rotarod test was used to evaluate motor coordination by testing the ability of rats to remain on a revolving rod. Rats were placed on a rotating drum with a revolving speed accelerating from 0 to 50 rpm and maintained at the final speed for 150s. The total time that the rat remained balanced on the rotating rungs was recorded for each trial. The rats were trained for 2 trials per day for the 3 days before surgery and tested for 3 trials per day on test days following TBI.

#### 2.4.3 Foot fault test

The foot fault test is sensitive to deficits in descending motor control. Rats were placed on a steel grid floor (20 × 40 cm with a mesh size of 2 cm^2^), which was elevated 1 m above the floor. Locomotive behavior was recorded by a 5‐min video. The total number of steps and the number of foot fault errors were counted. Fault foot error was defined as the misplacement of a forelimb or hindlimb such that it fell through the grid.

#### 2.4.4 Cylinder test

The cylinder test was employed to assess forepaw use and asymmetry. During the test, the rat was placed in a transparent cylinder (25 cm high, 15 cm diameter) and videotaped for 5 min. The number of forepaw (left/right/both) placements was recorded and analyzed off‐line. The relative proportion of right (ipsilateral) forepaw contacts were calculated as (right‐left)/(left + right + both) ×100%.

### 2.5 Immunofluorescence staining

Rats were terminally anesthetized with isoflurane and perfused with saline followed by 4% paraformaldehyde. Brains were removed and placed in a vial containing 4% paraformaldehyde for 4 h of fixation, then transferred to 20% and then 30% sucrose in PBS for cryoprotection. Brains were sliced on a sliding microtome. Immunostaining was performed on 30‐um free‐floating coronal sections. The sections were immunostained to detect the histological presence of myelin (MBP, Abcam), oligodendrocyte progenitor cells (Olig2, Millipore), and microglia (Iba‐1, Wako; CD206, R&D). The sections were incubated at 4°C overnight with the specific primary antibody, followed by incubation with fluorescence‐conjugated secondary antibodies (Jackson ImmunoResearch, West Grove, PA, USA, 1:500). Images were captured on an Olympus laser‐scanning confocal imaging system. All images were processed with Image J for cell‐based counting of automatically recognized Olig2, Iba1+, and CD206 + immunopositive cells. The mean was calculated from the three fields in the cortex, corpus callosum (CC), or striatum (Str) of each section and adjusted to express as mean number of cells per square millimeter.

### 2.6 TEM

Transmission electron microscopy was used to examine white matter ultrastructure following 28 days post‐CCI. Animals were treated as described previously.^8^ Briefly, affected tissue encompassing the CC was dissected into 1 mm^3^ tissue blocks and fixed in 2.5% glutaraldehyde for 5 day, followed by 1% osmium tetroxide for 1 h. After dehydration in alcohol series, tissues were embedded in 618# resin. Ultrathin sections were collected using a Reichert ultramicrotome, contrasted with uranyl acetate and lead citrate, and examined under a CM120 electron microscope at 80 kv. Analysis was performed on three images of randomly selected areas from three different rats per group. G‐ratio of myelinated axons was calculated by dividing the diameter of the axon without the myelin sheath by the diameter of the axon with the myelin health.^13^

### 2.7 RNA extraction and real‐time polymerase chain reaction (PCR)

At day 3, 7, or 14 days post‐CCI, rats were decapitated and brains were quickly removed, with the affected tissues surrounding the contusion site harvested. Total RNA was extracted by homogenization with Trizol reagent (Applied Biosystems, Grand Island, NY, USA). Reverse transcription was conducted using a RT reagent kit (Agilent, Santa Clara, CA, USA), and the reaction mixture was subjected to quantitative real‐time PCR. The primers used were as following: TNF‐α: 5′ CCCAGACCCTCACACTCAGATCAT 3′, 5′ CAGCCTTGTCCCTTGAAGAGAA 3′; TGF‐β: 5′ TGCGCTTGCAGAGATTAAAA 3′, 5′ CGTCAAAAGACAGCCACTCA 3′; CD32: 5′ AATCCTGCCGTTCCTACTGATC 3′, 5′ GTGTCACCGTGTCTTCCTTGAG3′; CD86: 5′ GACCGTTGTGTGTGTTCTGG 3′, 5′ GATGAGCAGCATCACAAGGA3′; iNOS: 5′ CAAGCACCTTGGAAGAGGAG 3′, 5′ AAGGCCAAACACAGCATACC 3′; GAPDH: 5′ AAGATGGTGAAGGTCGGTG3′, 5′ GTTGATGGCAACAATGTCC3′. CCL‐22:5′ CTGATGCAGGTCCCTATGGT 3′, 5′ GCAGGATTTTGAGGTCCAGA3′. Real‐time data were analyzed with a Mastercycler realplex analysis system (Eppendorf, Hauppauge, NY, USA). All samples were performed in triplicate. Thermal cycling condition was set according to the manufacturer's recommendations. Relative quantification of target mRNAs was normalized to GAPDH expression. Animals in the sham group were used as the calibration samples.

### 2.8 Statistical analysis

Statistical analyses were conducted using GraphPad Prism (GraphPad Software, La Jolla, CA, USA). All data were described by the mean ± standard error (SE). The normality was determined by visual inspection of their histograms, QQ Plots and Kolmogorov–Smirnov test. The difference in means between two groups was assessed by two‐tailed Student's *t*‐test. Differences in means among multiple groups were analyzed using 1‐ or 2‐way ANOVA with time or treatment as the independent factors. For all analyses, *p* < 0.05 was considered statistically significant.

## 3 RESULTS

### 3.1 EP improves neurobehavioral function following TBI

To confirm the CCI model, we first sought to examine sensorimotor functional outcomes in rats. CCI was executed on the right hemisphere and resulted in sensorimotor deficits as demonstrated by the cylinder, foot fault, hang wire and rotarod tests assessed over 21 days after TBI. As shown in Figure 1B–E, sham operation alone slightly changed the behavioral outcomes but recovered quickly after surgery. However, animals subjected to CCI showed markedly worse performance. CCI animals displayed increased asymmetric paw preference reflective of contralateral limb weakness or neglect throughout the testing period, with peak asymmetry occurring within the first 3 days following CCI (Figure 1B). Likewise, the number of errors on the foot fault test peaked in the initial several days following CCI, then steadily improved over the remaining test days, yet still remained significantly worse than sham rats throughout the timeframe tested (Figure 1C). Thus, although some spontaneous recovery occurred in descending motor control, TBI + Veh rats remained impaired 21 days following CCI in terms of locomotor accuracy.

In addition to the sensorimotor tests that reflect asymmetry or errors, we also wished to examine strength and balance/proprioception using the hang wire test and rotarod, respectively. Particularly in the initial week following injury, CCI injury worsened performance on the hang wire test, a measure of weakened neuromuscular strength and/or coordination to escape without falling (Figure 1D). A gradual recovery occurred over the remaining 2 weeks, but impairment was still detectable at 21 days following CCI. The rotarod requires intact balance and proprioceptive function to execute adaptive locomotion. Rats subjected to CCI displayed significantly attenuated total time on the rotating rod prior to falling in the week following injury (Figure 1E). After the initial week following CCI, the rats appeared to partially recover the ability to remain balanced on the rotating rod yet remained impaired compared with sham‐operated rats at 3–21 days post‐CCI.

Ethyl pyruvate treatment significantly improved performance on the rotarod following CCI, improved symmetry of limb use assessed by the cylinder test, improved performance on the hang wire test within 10 days post‐CCI, and markedly attenuated the number of foot faults up to 21 days post‐CCI (Figure 1B–E). Similar to TBI + Veh rats, TBI + EP rats demonstrated recovery of functional behaviors following the initial week after CCI. Notably, the progression of recovery appeared to be initiated at an earlier time point compared to TBI + Veh rats, as indicated by the asterisks in Figure 1. These results indicate that EP treatment significantly improved long‐term sensorimotor functions impaired by CCI.

### 3.2 EP confers long‐term preservation of WM after TBI

**FIGURE 2:**
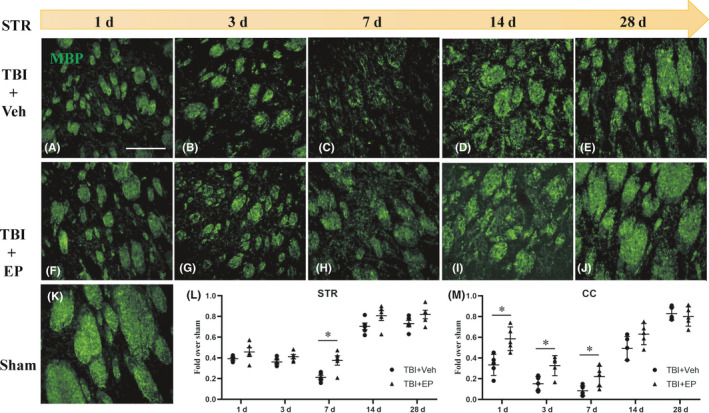
Effect of EP treatment on myelin loss after TBI. (A–K) Representative images of MBP staining in white matter at different time points after TBI (MBP: green). (L, M) show the fluorescence intensity of MBP (+) in the corpus callosum (CC) and striatum (Str), respectively. Scale bar = 100 µm, *n* = 5 animals/group. *p < 0.05 vs. vehicle‐treated TBI animals

We examined overt damage to the myelin sheath in the Strand CC by assessing the loss of myelin basic protein (MBP) immunostaining. Myelin loss in striatal fiber bundles began at the acute stage following CCI and became progressively more disorganized through day 7 post‐CCI in vehicle‐treated rats (Figure 2A–C,L). By 14 days and continuing through 28 days post‐CCI, myelin immunostaining intensity increased toward sham levels (Figure 2L), and striatal MBP + structures appeared more organized (Figure 2D,E). Treatment with EP improved myelin immunostaining at 7 days following CCI (Figure 2F–H,L) and robustly increased MBP immunostaining and organization of fiber bundles 14–28 days following CCI (Figure 2I,J). Sham MBP immmunostaining is shown for reference (Figure 2K). Semi‐quantification of MBP signal intensity is presented in Figure 2L. Similar results were observed in CC sections, wherein treatment with EP attenuated the loss of MBP immunostaining over the initial 7 days following CCI (Figure 2M). Similar to vehicle‐treated CCI brain, robust recovery of MBP immunostaining was observed 14–28 days following CCI in EP‐treated brain.

**FIGURE 3:**
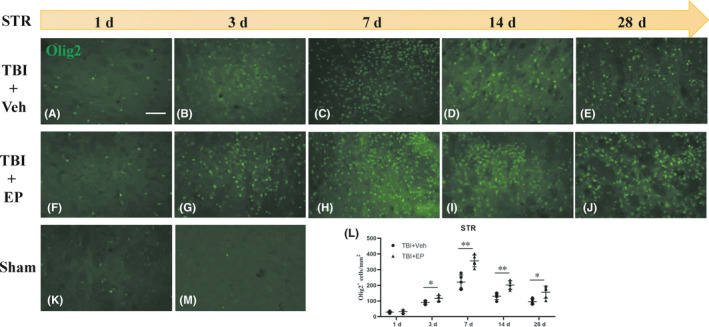
Changes in number of OPCs following CCI. (A–K) Representative images of OPCs in striatum up to day 28 post‐CCI (Olig2: green).(M) Negative staining image for Olig2. (L) Quantification of Olig2^+^ cells as counted number per area (mm^2^). Scale bar = 100 µm, *n* = 5 animals/group. *p < 0.05, **p < 0.01 vs. vehicle‐treated TBI animals

The observation that MBP immunostaining appeared to improve over extended recovery times (14–28 days) led to the hypothesis that an increase in the number of oligodendrocyte progenitor cells (OPCs) may become available for remyelination within or prior to this time point. In order to study the regeneration of myelin after TBI in adult rats, immunofluorescence staining against Olig2, a marker of OPCs, was performed at 1, 3, 7, 14, 28 days post‐CCI. As shown in Figure 3, we observed an increased number of Olig2^+^ cells in the CC (not shown) and Str in tissue surrounding the lesion. Few OPCs were present in sham brain, scattered among brain regions. The number of Olig2^+^ cells in the STR proximal to the lesion increased continuously after injury and peaked at 7 days post‐CCI. In EP‐treated TBI rats, the number of Olig2^+^ cells at 3–28 days post‐CCI was markedly elevated compared with the vehicle‐treated group. These data support the concept that remyelination may be possible following TBI *via* the increased number of OPCs, and that EP treatment may stimulate white matter recovery by diminishing injury to the myelin sheath and/or stimulating OPC presence and remyelination.

**FIGURE 4:**
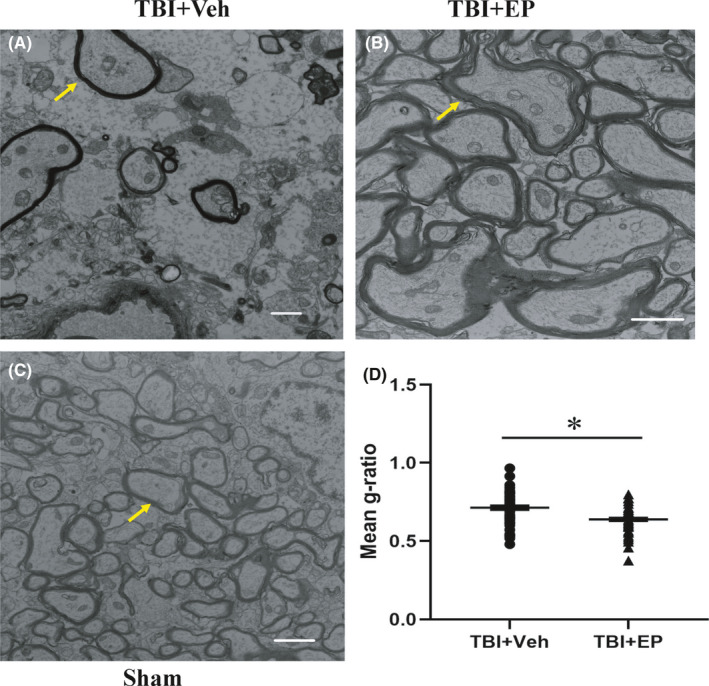
Ethyl pyruvate treatment improved white matter remodeling after TBI. Representative EM images from corpus callosum of a vehicle‐treated TBI brain (A), EP‐treated TBI brain (B), and sham‐operated brain (C) at day 28 post‐CCI. The arrows indicate axons covered by myelin. (D) EP treatment reduced the g‐ratio compared to the vehicle treatment. 62 myelinated axons per group were analyzed (*n* = 62). Scale bars = 1 μm. *p < 0.05 vs. vehicle‐treated TBI animals. The images are from three brains per group with similar results

To further examine ultrastructural changes in WM and how EP may confer protection, we processed brains for TEM at 28 days post‐TBI. The myelin sheath of CC was intact and arranged compactly and regularly in the sham surgery group (Figure 4C). Compared with sham, vehicle‐treated CCI brain exhibited a qualitatively decreased presence of myelinated axons and decreased thickness of myelin sheath (Figure 4A). However, EP treatment increased the number of myelinated axons and reduced the g‐ratio 28 days post‐TBI (Figure 4B,D).

### 3.3 EP shifts microglia polarization toward the M2 phenotype and mitigates cerebral inflammation following TBI

**FIGURE 5:**
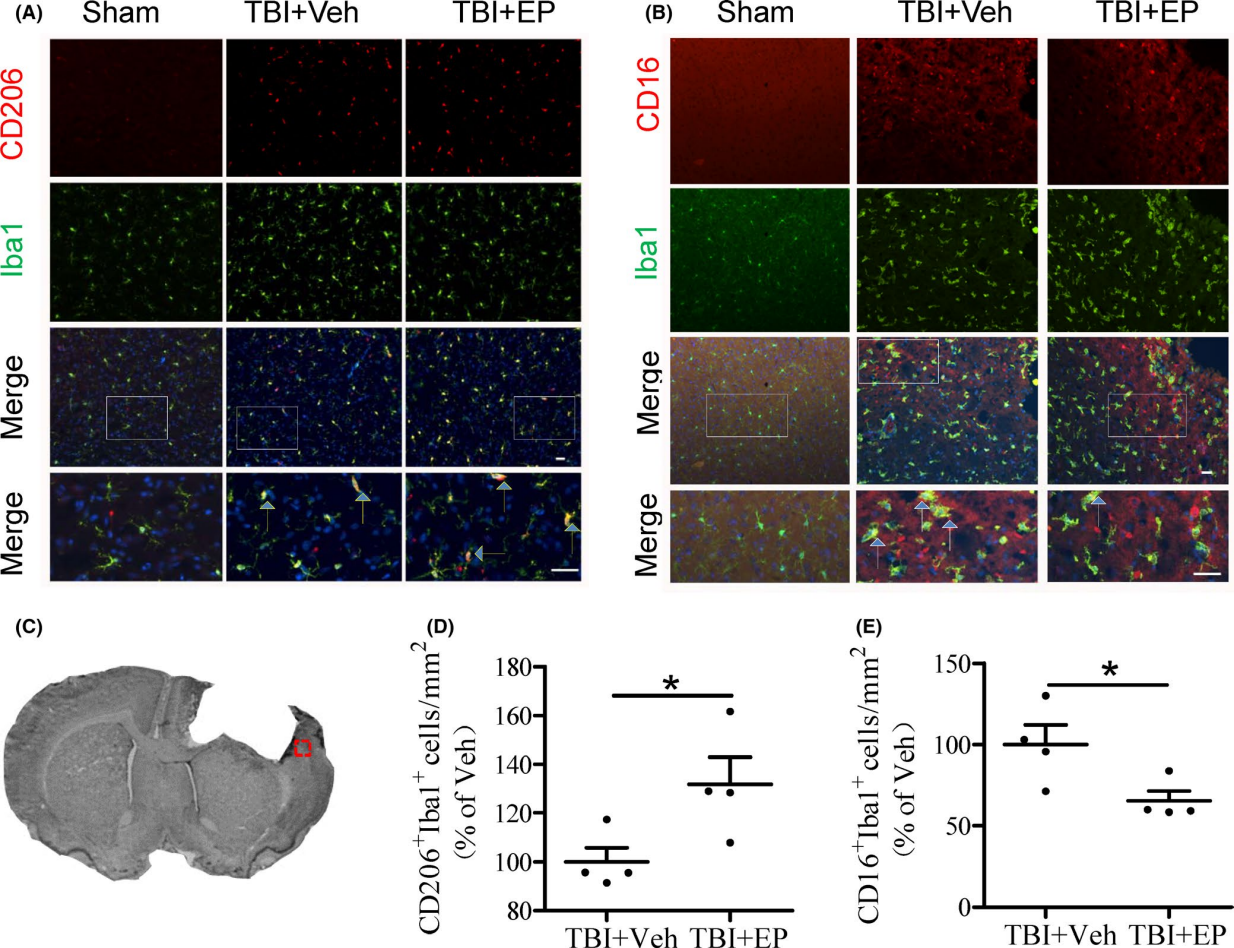
Ethyl pyruvate modulated the polarization of microglia after TBI. (A–B) Representative images of Iba‐1/CD206 (A) or Iba‐1/CD16 (B) immunohistochemistry in impaired cortex border (CTX: cortex) at day 3 post‐CCI. Iba‐1: green, CD206: red, DAPI: blue, yellow arrow: Iba‐1 (+) CD206 (+) microglia, white arrow: Iba‐1 (+) CD16 (+) microglia. Scale bar = 10 µm. (C) The empty square box depicts the region of interest relevant to the peri‐contusion border, from where tissues were sampled for immunohistochemical images. (D–E) Quantification of CD206+/Iba‐1 + or CD16+/Iba‐1 + cells in the cortex. Data are expressed as percentages of cell numbers vs. vehicle‐treated TBI brains. *p < 0.05, *n* = 4 animals/group

To examine whether EP modulates microglia polarization, we performed co‐immunofluorescence staining for both the microglia/macrophage marker Iba‐1 and the M2‐associated marker CD206 or M1‐associated marker CD16 in the affected cortex at 3d after TBI. Expression of Iba‐1 increased continuously after CCI injury in both vehicle‐ and EP‐treated brain (Figure 5), indicating the mobilization of microglia/macrophage to the injured tissue. Compared with vehicle‐treated CCI brain, co‐localization of CD206 expression with Iba‐1 positive cells significantly increased in EP‐treated brains post‐CCI. On the contrary, the percentage of CD16^+^ M1 microglia decreased in EP‐treated brains compared with the Vehicle‐treated group. Together these data indicate that the microglia/macrophage response to injury appears to be active in both vehicle‐ and EP‐treated TBI brain, but EP treatment stimulates the M2 phenotype in microglia/macrophage.

**FIGURE 6:**
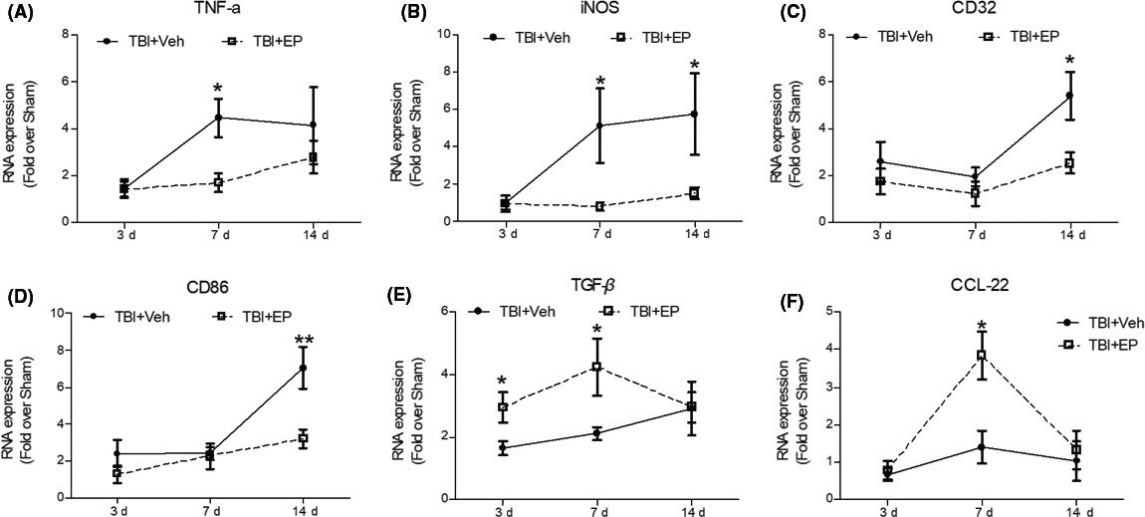
Ethyl pyruvate modulated the level of inflammatory‐related factors after TBI. (A–D) Real‐time PCR evaluation of the pro‐inflammatory molecules TNF‐α, iNOS, CD32, and CD86 in TBI rats 3–14 days post‐injury. (E–F) Real‐time PCR measurements of the antiinflammatory molecules TGF β and CCL‐22 in TBI rats 3–14 days post‐injury. Data are mean ± SE, *n* = 5 animals/group. *p < 0.05, **p < 0.01 vs. vehicle‐treated TBI animals

M2 polarization of microglia/macrophage is associated with an antiinflammatory state, reflected by diminishing pro‐inflammatory factors and increased presence of antiinflammatory molecules. Six molecules related to modulation of inflammation (CD32, CD86, CCL‐22, iNOS, TNF‐α, TGF‐β, and CCL‐22) were validated by real time‐PCR (Figure 6). The expression of proinflammatory markers, including CD32, CD86, iNOS, and TNF‐α, was markedly increased in vehicle‐treated TBI animals, and these markers were significantly suppressed in EP‐treated TBI groups at 7 or 14 days after CCI. Furthermore, levels of antiinflammatory molecules, including TGF‐β and CCL‐22, slightly increased after CCI, but were further elevated by EP treatment, most notably at 7 days post‐CCI.

## 4 DISCUSSION

Recent studies have shown that EP can attenuate injury in cerebral ischemic and traumatic injury models, perhaps by increasing energy metabolites, inhibiting oxidative and neuroinflammatory, and suppressing apoptosis.^8^, ^10^, ^14^, ^15^ However, whether EP can confer protection of white matter or improve white matter recovery had not yet been examined. We demonstrate here that, in addition to improved behavioral outcomes afforded by post‐TBI EP treatment, EP treatment alleviates WMI and is associated with increased polarization of microglia/macrophage to an M2 phenotype.

Demyelination and axonal damage are important processes in the pathogenesis of TBI. Our data show that EP attenuates TBI‐induced myelin loss and improves myelin regeneration, which may decrease axon loss and lead to neurological functional recovery. In accordance with our study, protective effects of EP were reported previously in models of peripheral nerve degeneration.^16^, ^17^ EP regulated the expression of the myelin‐related transcription factor, c‐Jun, and inhibited axonal degradation during Wallerian degeneration.^17^ In addition, EP inhibited oxidative stress and pro‐inflammatory cytokines, and exerted powerful protective effects on axonal damage and demyelination *in vitro*.^18^

Promotion of white matter remodeling by EP following injury can be stimulated at several points. A recent study found that EP promotes the generation of OPCs and mitigates histological demyelination in a cuprizone (CPZ)‐induced mouse demyelination model.^19^ In the same study, EP treatment induced M2‐like polarization of microglia/macrophage. Our data also support the hypothesis that EP may function to improve recovery from TBI by OPC generation and possible subsequent differentiation, as well as to promote M2 polarization of microglia/macrophage. The role of M2 microglia/macrophage in recovery from WMI is still under investigation, particularly in TBI models.^20^ However, the phagocytic activity of microglia/macrophage that is associated with the M2 phenotype can be highly beneficial in removing toxic elements, such as myelin fragments, from the extracellular milieu.^21^, ^22^, ^23^, ^24^ This “clean up” action of microglia/macrophage might then also facilitate the differentiation and targeting of OPCs to demyelinated axons for recovery and repair of myelinated tracts.^25^, ^26^

Influencing microglia/macrophage (please add/macrophage here) polarization to the M2 phenotype has been proposed as a possible strategy in the treatment of neurological disorders.^27^, ^28^, ^29^, ^30^, ^31^ In this study, we found that the protective effect of EP on WMI may be associated with microglia polarization toward M2 and subsequent increase in the expression of antiinflammatory related molecules and suppression of pro‐inflammatory factors. As a potent antiinflammatory molecule, EP has been reported in multiple studies to suppress the release of various pro‐inflammatory factors in experimental models of cerebral injury.^8^, ^19^, ^32^, ^33^ In this study, we not only examined the pro‐inflammatory factors released by M1‐like microglia (CD32, CD86, iNOS, and TNF‐α), but also assessed those released by M2‐like microglia, including TGF‐β and CCL‐22. This possible association between microglia/macrophage polarization and WMI raises the potential that stimulation of M2 polarization may become a new target for therapeutic intervention in demyelinating diseases.

Ethyl pyruvate has undergone clinical safety trials and is considered safe at therapeutically relevant doses. In addition to the highlighted studies in brain, EP was reported to be protective against peripheral nerve degeneration in Schwann cells.^17^ Thus, EP may be a promising candidate for demyelination diseases not only in central nervous system (CNS), but also in peripheral nervous system. Additional studies to determine potential mechanistic actions of EP in injured white matter is merited and will likely yield potential targets in the development of therapeutic interventions.

## 5 CONCLUSIONS

Taken together, the present study suggests a therapeutic effect of EP on WMI induced by TBI in addition to the improvement of behavioral outcomes. Thus, EP may provide a new clinical strategy for improving neurological functional recovery after TBI.

## CONFLICTS OF INTEREST

The authors declare no conflict of interest. The funders had no role in the design of the study; in the collection, analyses, or interpretation of data; in the writing of the manuscript, or in the decision to publish the results.

## Data Availability

Data sharing not applicable—no new data generated.
